# Impact of COVID-19 on chronic pain patients: a pain physician’s perspective

**DOI:** 10.2217/pmt-2020-0035

**Published:** 2020-08-10

**Authors:** Saba Javed, Joey Hung, Billy K Huh

**Affiliations:** ^1^Department of Anesthesiology, University of Texas Health Science Center at Houston, Houston, TX 77030, USA; ^2^Department of Anesthesiology, University of Texas MD Anderson Cancer Center, Houston, TX 77030, USA

**Keywords:** chronic pain, opioids, pain management

26 October 2017 and 1 March 2020, two important dates that will be remembered for years to come, former signifying the declaration of opioid crisis as a public health emergency in the USA and latter when coronavirus disease 2019 (COVID-19) was declared a pandemic in the USA [1]. Since 1999, more than 750,000 have people died due to the opioid crisis and, as of May 2020, over 90,000 Americans’ lives have succumbed to the COVID-19 pandemic [2]. Two inherently distinct crises but ultimately unified with the commonality of creating suffering and death. It is obvious that COVID-19 has impacted all aspects of the human existence, particularly the healthcare arena including the patients, and more so the chronic pain patients. The full impact of the pandemic on this sub-population and ultimately the opioid crisis will reveal itself in the years to come, however at this juncture, it is critical to manage the needs of our patients and continue to provide physicians and other therapeutic access in traditional and nontraditional ways.

## Discussion

Just like most other medical specialties, the field of chronic pain is one of the hardest hit from the COVID-19 pandemic leaving many patients over burdened with their chronic pain and their on-going treatment delayed. Over the past few months, in order to mitigate the spread of COVID-19, in-person access to physicians was limited and predominantly moved to telemedicine. While telemedicine has been beneficial in maintaining contact with patients and continuing therapy, due to an inability to perform a physical exam, it did delay and has created lag in proper diagnosis and treatment. Furthermore, diagnostic imaging such as x-rays, CT scans, MRI scans all have had to be deferred, which while certainly not required to make the diagnosis can be beneficial for diagnostic and therapeutic purposes. Additionally, due to deferment of elective cases, patients who were currently awaiting to undergo extensive surgery such as laminectomy, discectomy, hip arthroplasty or total knee arthroplasty have had their surgeries delayed and are having their pain managed with medicines, in some cases narcotics. Interventional pain procedures that needed multiple trials such as medial branch blocks, spinal cord stimulator trials have also been hindered further postponing the subsequent therapeutic procedures like radiofrequency ablations and spinal cord stimulator implants.

Due to the on-going pandemic, other sources of stressors and pain have amplified ultimately worsening chronic pain. For instance, physical therapy (PT) programs, which are an integral part of multimodal chronic pain management, have been halted due the COVID-19 pandemic [3]. Numerous patients who have had positive outcomes with home PT, land-based PT or aquatherapy have been unable to receive their therapy sessions resulting in potentially worsened pain. Also part of the multimodal pain treatment strategy is the assistance of psychiatrist, psychologist and pain counselors to assist with cognitive behavior therapy, coping skills and managing stress. And with their offices practicing social distancing with appointment deferred, new and on-going treatment has been further delayed for chronic pain patients. Additionally, it is also well known that psychosocial issues can lead to worsened pain perception; hence, it comes as no surprise that this pandemic has caused psychological stress worsening chronic pain symptoms [4,5]. Stresses like financial loss, personal loss, anxiety etc., all can take a toll on a patient’s chronic pain state further exacerbating their symptoms. Thus, with very little ability to use a multidisciplinary approach to pain management, pain physicians have had very limited therapeutic options, including prescribing opioids to keep patients away from the already overburdened emergency rooms.

On 19 March 2020 governor Greg Abbott approved the Texas Medical Board to temporarily suspend Title 22, Chapter 174.5 (e2A) of the Texas Administrative Code [3]. This waiver allowed for temporary telephone refills of valid prescriptions for treatment of chronic pain including narcotic medications. This motion was groundbreaking especially with the seriousness of the opioid crisis in the USA. This has meant that chronic pain physicians are able to prescribe narcotics and refills over the phone through telemedicine. With the potential for over prescription of narcotics, this could further exacerbate the opioid crisis in the USA. In our practice alone, prescription narcotics were increased by 14% in March and up by 20% in April as compared with February. Studies have shown that long-term exposure to opioids can lead to opioid misuse subsequently causing addiction and diversion to other people without a prescription [6]. In addition to slight over prescription, there are also patients who were in the process of being weaned from opioids. They are experiencing hindrance in their weaning process, and in some cases a halt, further adding to the opioid use.

Currently, as different states start to reopen and as we resume elective procedures, we have been noticing a backlog of patients to be seen that are both established and new consults. Patients have had their appointments rescheduled and are unable to be seen until weeks or months later. Resumption of elective procedures still means some patients may have to wait weeks to months for their procedures. Moreover, patients are still apprehensive about proceeding with elective procedures, and rightfully so, at the risk of being exposed to COVID-19. Additionally, many patients who initially had medical insurance lost their coverage as they were either furloughed or laid off by their job. This has already hindered and could further delay interventional or therapeutic treatment plans. Furthermore, with new rules being implemented in hospitals, surgery centers and clinics to avoid viral spread, this has and will result in increased turn-over time, limiting the number of procedures physicians can safely do in a day. This ultimately would cause patients to receive interventions at a later time which could prolong their chronic pain symptoms, ultimately putting pain physicians at a precarious position of managing pain with opioids.

However, as discussed above, there is a silver lining from this pandemic, and that is the implementation of telemedicine [7]. As physicians navigate through this pandemic and try to maintain contact with their patients, telemedicine is likely here to stay even after all the dust settles. It has been a great tool in mitigating virus spread and still has a significant impact in helping patients with maintaining their chronic pain regimen. It helps patients get care in distant areas. Telemedicine is also helpful in patients who were doing well with their current pain regimen simply wanting refills.

## Conclusion

The COVID-19 pandemic has impacted everyone around the world including all sectors of the economy, all businesses, healthcare industry, physicians and patients alike. With the deferment in elective procedures to contain the spread of COVID-19, limited access to multimodal therapeutic modalities such as PT, psychologist and psychiatrist appointments and with the added psychosocial emotional stressors from the pandemic, economic and financial insecurities, the potential for worsened chronic pain symptoms are very high. And with limited availability of multimodal therapeutic alternatives employed by pain physicians, these physicians are having to prescribe pain medications until the patient is able to get their treatment. Chronic pain physicians are having to walk the fine line of balancing the previously on-going opioid crisis and now this pandemic in managing the pain with the limited resources available. The key moving forward is trying to closely follow these patients, wean opioids and get them back on their pre-COVID-19 treatment plan as other multimodal therapeutic options open up.
